# Scleral Cross Section Area and Volume and Axial Length

**DOI:** 10.1371/journal.pone.0093551

**Published:** 2014-03-28

**Authors:** Jost B. Jonas, Leonard Holbach, Songhomitra Panda-Jonas

**Affiliations:** 1 Department of Ophthalmology, Medical Faculty Mannheim, Heidelberg University, Mannheim, Germany; 2 Department of Ophthalmology, Friedrich-Alexander University Erlangen-Nürnberg, Erlangen, Germany; Cardiff University, United Kingdom

## Abstract

**Purpose:**

To examine whether the scleral cross sectional area and estimated scleral volume are associated with a longer axial length in human eyes.

**Methods:**

Histologic anterior-posterior sections running through the pupil and the optic nerve head were examined. Using a light microscope, we measured the thickness of the sclera at the limbus, ora serrata, equator, midpoint between equator and posterior pole (MPEPP), peripapillary region and posterior pole. Additionally we determined the length and the cross section area of the sclera.

**Results:**

The histomorphometric study included 214 human globes of 214 subjects (mean age: 62.5±13.9 years) (147 eyes enucleated due to malignant choroidal melanoma or due to other non-glaucomatous reasons; 67 eyes enucleated due to secondary angle-closure glaucoma). Mean axial length was 25.1±1.8 mm (median: 24.0 mm; range: 20–35 mm). Scleral thickness measurements decreased with increasing axial length for values taken at the equator (*P* = 0.008; correlation coefficient r = −0.18), MPEPP (*P*<0.001;r:−0.47), optic nerve head border (*P*<0.001;r = −0.47) and posterior pole (*P*<0.001;r = −0.54). Scleral cross section area decreased significantly with increasing axial lengths for the regions at or behind the equator (*P* = 0.002;r = −0.21), at or behind the MPEPP (*P* = 0.001;r = −0.25), and at or behind the optic nerve head border (*P* = 0.001;r = −0.24). Scleral volume measurements were not significantly associated with axial length

**Conclusions:**

Despite an associated increase in surface area, eyes with longer axial length do not have an increase in scleral volume. It may point against a scleral volume enlargement to play a role in the process of axial elongation.

## Introduction

Size and shape of the eye are markedly determined by the sclera as the outer firm layer of the ocular globe. The constancy of the globe shape is of high importance for the ocular optical system which profoundly depends on constant distances between the cornea, lens and retina. An elongation of the axial length of the eye leads to axial myopia which has become one of the major causes of visual field defects, visual impairment and blindness worldwide [1]–[4]. The importance of myopia will further increase due to the myopic shift taking place in particular in Asian countries at the Pacific rim [5]–[7]. Previous studies, as early as by Heine in 1899, have shown that axial elongation is associated with thinning of the sclera in the region at or behind the equator. This tissue thinning was more marked closer to the posterior pole [8]–[11]. These studies left unclear whether axial elongation only leads to a thinning of scleral tissue, e.g. in a process of remodeling of available scleral tissue, or whether axial elongation is also associated with an increase in scleral tissue volume, suggesting an additional new formation of scleral tissue. The question whether axial elongation is associated with an increase in scleral volume can be addressed by measuring the scleral cross section area and volume in eyes of different axial length. Both parameters, however, have not been determined nor have associations of these two parameters with axial length been assessed in previous studies. Answering the question could be of interest for the discussion of the mechanism of axial elongation or myopization of the globe. We therefore performed this histomorphometric study on human globes with different axial length, measured the globe diameters and scleral thickness, determined scleral cross section area and volume, and correlated the scleral parameters with axial length.

## Methods

### Ethics Statement

The Medical Ethics Committee II of the Medical Faculty Mannheim of the Ruprecht-Karls University Heidelberg approved the study protocol. In agreement with the approval by the ethics committee, informed consent was not obtained since the globes had been enucleated up to 30 years before the study was initiated.

The study included eyes of Caucasian patients. The globes had been enucleated mostly due to painful secondary angle-closure glaucoma with complete or almost complete loss of vision or because of malignant choroidal melanomas. Eyes with congenital glaucoma were excluded. In the glaucomatous group, vision was completely or almost completely lost. At the time when the eyes with malignant tumors were enucleated, no other treatment modalities were available or were thought not to be suitable for tumor removal with respect to its location and size. Most of the eyes had been included in previous studies on different topics [11], [12]. In a standardized manner immediately after enucleation, the globes were fixed in a solution of 4% formaldehyde and 1% glutaraldehyde. They remained in the fixative at room temperature for one week. Using anatomical landmarks such as the insertion of the oblique muscles, the 12 o'clock position of the globes was marked at the limbus. The axial length and the horizontal and vertical diameters of the globe were measured with a ruler or caliper. The globes were then processed for histological sectioning as follows: The direction of the histologic cut depended on the location of the tumor in the group of eyes with a malignant choroidal melanoma; it was horizontal in the glaucoma group. Otherwise the preparation of the globes did not vary between the glaucomatous eyes and the non-glaucomatous eyes or between the axially elongated and the non-axially elongated eyes. The globes were prepared in routine manner for light microscopy. A section running through the pupil and the optic nerve head was cut out of the fixed globes. The segments were dehydrated in alcohol, imbedded in paraffin, sectioned for light microscopy, and stained by the Periodic-Acid-Shiff (PAS) method or with hematoxycilin eosin. For all eyes, one section (thickness: 8 μm) running through the central part of the optic nerve head was selected for further evaluation.

**Figure 1:**
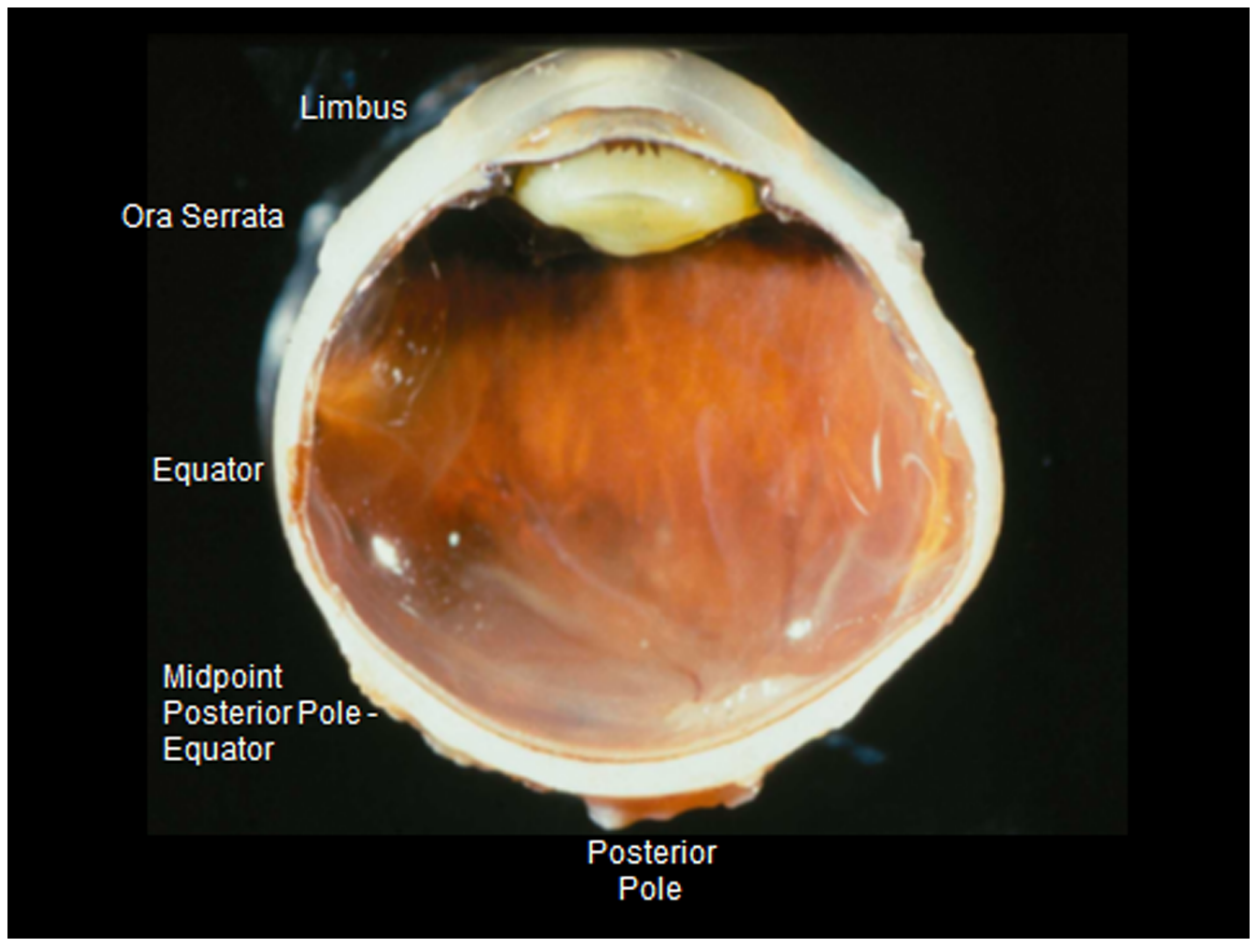
Photograph showing the measurement points of scleral thickness.

**Figure 2:**
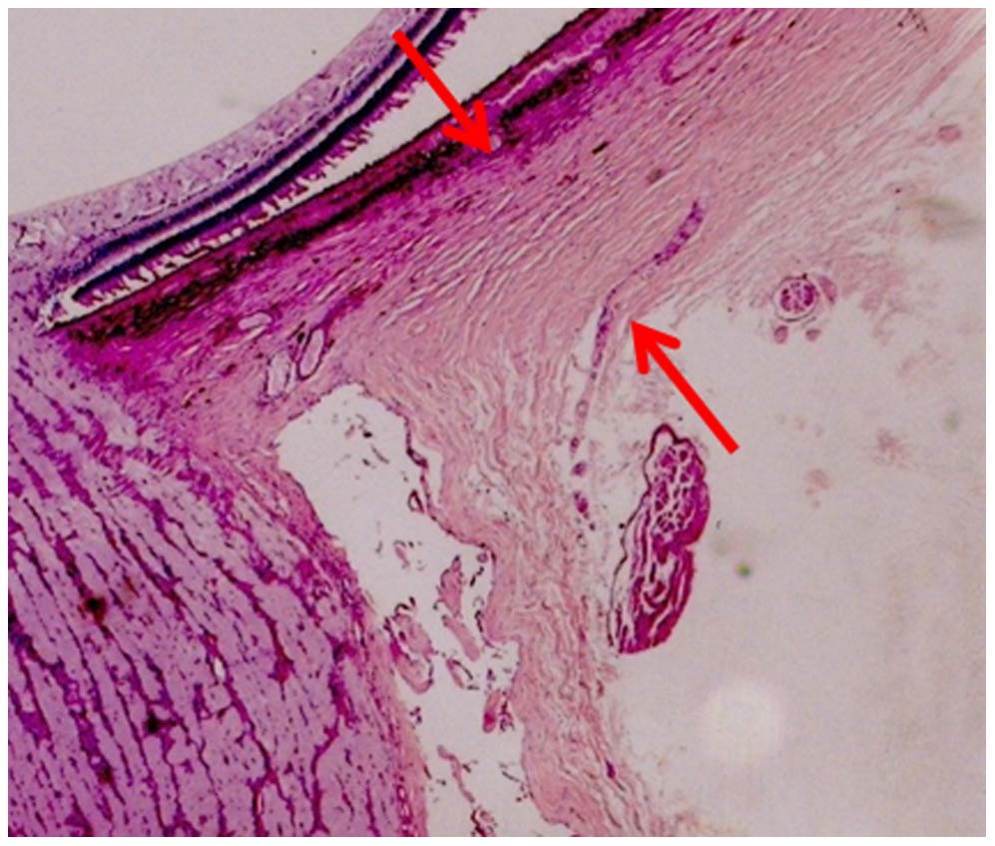
Histo-photograph showing the location of the scleral thickness measurement (between the two red arrows) at the merging point of dura mater with the posterior sclera.

Using a light microscope with an in-built millimeter scale, the globes were histomorphometrically examined and measured by the same examiner (JBJ). Measurement points for assessing the thickness of the sclera were the limbus, ora serrata, equator, midpoint between the posterior pole and the equator (MPEPP), the site just outside of the merging point of the optic nerve dura mater with the sclera, and the posterior pole (Fig. 1, 2). Using calipers, the axial length was measured after the globes were fixed in formaldehyde and glutaraldehyde, usually before opening and cutting the globes. Since all the globes were prepared in the same ophthalmic-pathological laboratory in which the routine measurements of the globe diameters had not changed during the study period, the axial length of the globes included into the study was measured with the same technique. The technique has been described in detail already in previous studies [11]–[13].

Statistical analysis was performed using SPSS for Windows, version 21.0 (IBM-SPSS, Chicago, Illinois, USA). In a first step, we determined the mean values and the standard deviations. In a second step, we calculated the mean of all scleral thickness measurements at and behind the equator, at or behind the ora serrata, and at and behind the limbus, respectively. We also calculated the globe circumference using the sagittal diameter as reference and the formula of a sphere. We multiplied the mean scleral thickness with the globe circumference corresponding to the respective region (at and behind the equator, at or behind the ora serrata, and at and behind the limbus) and arrived at the scleral cross sectional area in the respective region. In a third step, we determined the mean globe radius as the mean of axial length, horizontal globe diameter and vertical diameter, and calculated the globe surface. We multiplied the surface value for the respective region with the mean scleral cross sectional area in the respective region to arrive at the scleral volume for the respective region. Finally, we assessed potential associations between scleral cross sectional area and scleral volume with axial length. The level of significance was 0.05 (two-sided) in all statistical tests.

The assessment of the globe circumference was tested on 10 globes the sagittal diameter and circumference of which was measured using the ImageJ program (available at: http://rsbweb.nih.gov/ij/download.html). The sagittal diameter and the circumference of 10 globes of different shape were measured and the measured globe circumference was compared with the calculated globe circumference using the sagittal globe diameter and the formula of a circle. The assessment of the globe volume was partially examined by measuring the area, the sagittal diameter and the diameter perpendicular to the horizontal diameter on the histologic slides 10 globes of different shape using the Image J program.

## Results

The study included 214 human globes of 214 subjects with a mean age of 62.5±13.9 years. There were 147 eyes enucleated due to malignant choroidal melanoma or due to other non-glaucomatous reasons, and 67 eyes enucleated because of secondary angle-closure glaucoma. Mean axial length was 25.1±1.8 mm, horizontal globe diameter was 23.7±1.4 mm, and vertical globe diameter was 23.7±1.4 mm (Table 1).

**Table 1:** Size measurements of the globes.

Parameter	Mean	Median	Range
Age (Years)	62.5±13.9	64	24–89
Axial Length (mm)	25.1±1.8	24.0	20.0–35.0
Horizontal Globe Diameter (mm)	23.7±1.4	24.0	20.0–29.0
Horizontal Globe Diameter (mm)	23.7±1.4	24.0	21.0–29.0

Mean cross sectional area of the sclera was for the region behind the limbus 39.7±6.2 mm^2^, for the region at and behind the ora serrata 31.2±6.2 mm^2^, for the region at or behind the equator 31.0±6.8 mm^2^, for the region at or behind the MPEPP 14.6±3.6 mm^2^, and for the region between the optic nerve border and the posterior pole 2.7±0.7 mm^2^ (Table 2):

**Table 2:** Measurements of the scleral cross sectional area (mm^2^) and estimated scleral volume (mm^3^).

Region	Mean	Median	Range
Scleral Cross Sectional Area (mm^2^)			
At or Behind the Limbus to Posterior Pole	39.7±6.2	39.1	25.4–57.4
At or Behind the Ora Serrata	31.2±6.2	31.3	17.2–52.4
At or Behind the Equator	31.0±6.8	31.5	9.3–51.4
At or Behind the Midpoint between Equator and Posterior Pole	14.6±3.6	14.9	3.2–25.4
Between Merging of Dura Mater with Posterior Sclera and Posterior Sclera	2.7±0.7	2.7	0.6–4.5
Scleral Volume (mm^3^)			
At or Behind the Limbus to Posterior Pole	987±163	987	693–1437
At or Behind the Ora Serrata	757±152	745	431–1265
At or Behind the Equator	615±138	617	195–1034
At or Behind the Midpoint between Equator and Posterior Pole	175±43	176	40–307
Between Merging of Dura Mater with Posterior Sclera and Posterior Sclera	31±8	31	7–57

Mean volume of the sclera was for the region behind the limbus 987±163 mm^3^, for the region at and behind the ora serrata 757±152 mm^3^, for the region at or behind the equator 615±138 mm^3^, for the region at or behind the MPEPP 175±43 mm^3^, and for the region between the optic nerve border and the posterior pole 31±8 mm^3^ (Table 2).

All scleral measurements were not significantly associated with gender nor age (all *P*>0.10). All mean scleral thickness values were highly significantly associated with increasing axial length (all *P*<0.001). Taken the individual scleral thickness measurements, the associations with axial length were not significant for the measurements taken at the limbus (*P* = 0.64) and at the ora serrata *P* = 0.90), while the scleral thickness measurements decreased with increasing axial length for the values taken at the equator (*P* = 0.008; r: −0.18), at the MPEPP (*P*<0.001; r: −0.47), optic nerve head border (*P*<0.001; r: −0.47) and posterior pole (*P*<0.001; r: −0.54).

**Figure 3:**
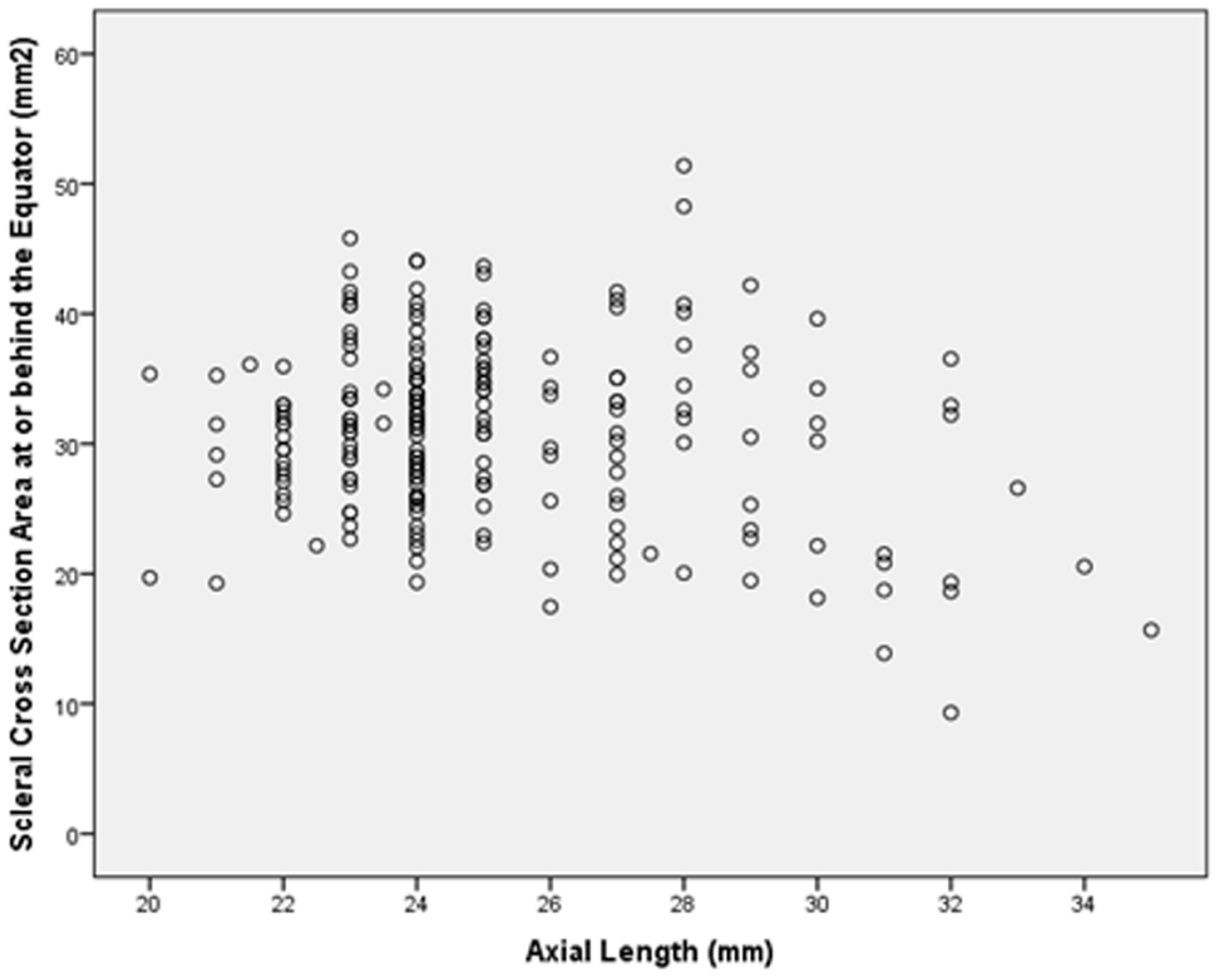
Scatterplot showing the distribution of the scleral cross section area at or behind the equator and axial length; the association was statistically significant (*P* = 0.002; correlation coefficient r: −0.21).

The scleral cross section area measurements decreased significantly with increasing axial lengths for the regions at or behind the equator (*P* = 0.002; correlation coefficient r: −0.21) (Fig. 3), for the region at or behind the MPEPP (*P* = 0.001; r: −0.25), and for the region at or behind the optic nerve head border (*P* = 0.001; r: −0.24). The scleral cross section area measurements taken at or behind the limbus (*P* = 0.88) or taken at or behind the ora serrata (*P* = 0.07; r: −0.13) were not statistically significantly associated with axial length.

**Figure 4:**
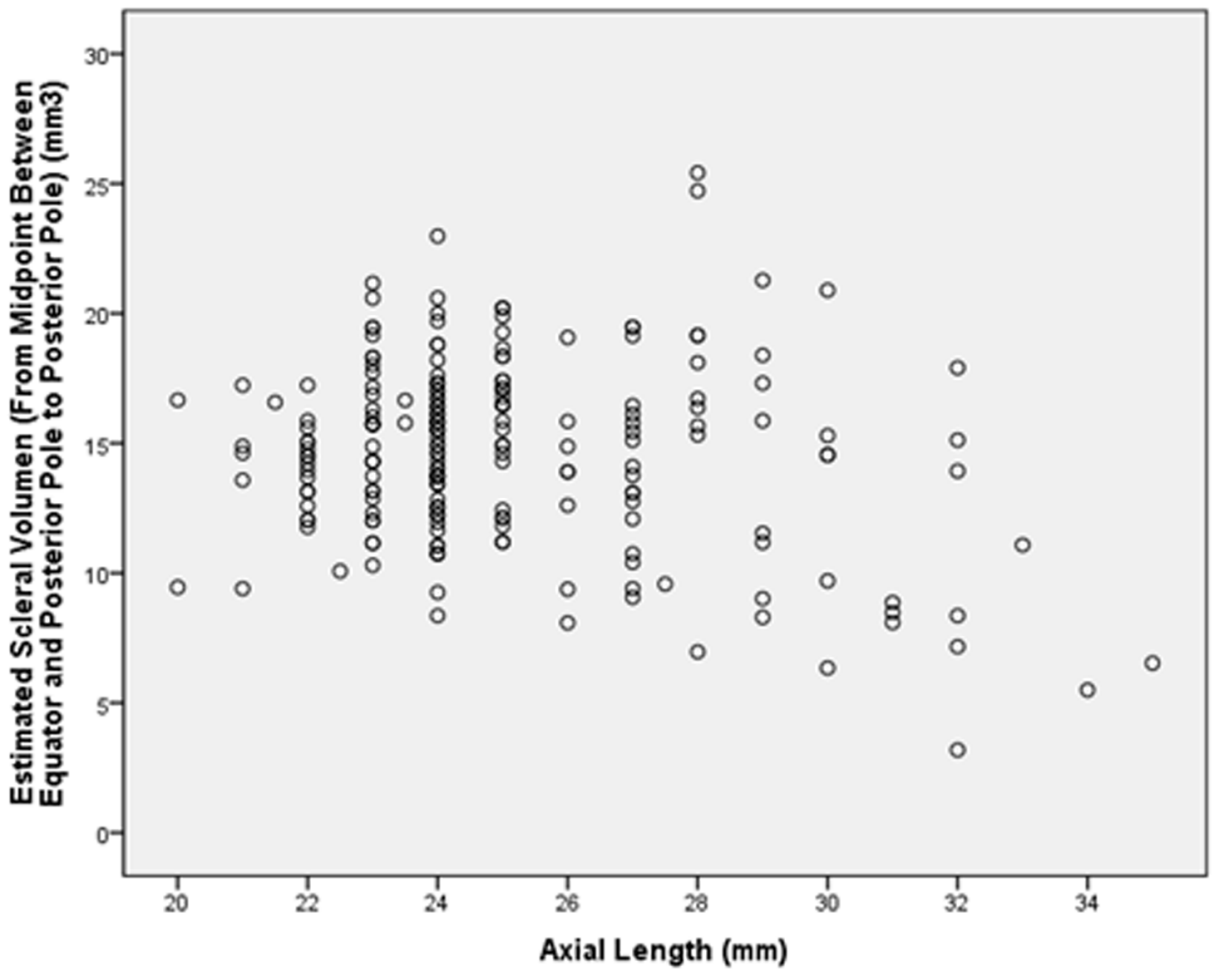
Scatterplot showing the distribution of the scleral volume at or behind the midpoint between equator and posterior pole and axial length; the association was not statistically significant (*P* = 0.12; correlation coefficient r: −0.14).

The scleral volume measurements showed a slight tendency towards decreasing values with increasing axial length; these associations were however not statistically significant (region at or behind the limbus: *P* = 0.61; region at or behind the ora serrata: *P* = 0.96; region at or behind the equator: *P* = 0.39; region at or behind the MPEPP: *P* = 0.12; r: −0.14 (Fig. 4); region at or behind the optic nerve head border: *P* = 0.10; r: −0.15).

The assessment of the method for determination of the globe circumference showed that the calculated values differed by a mean factor of 5.1±3.7% from the measured values of circumference. In a similar manner, the calculated area measurements differed by a mean factor of 4.0 ± 4.4%for the measured values of area.

## Discussion

In our histomorphometric study, scleral thickness measurements decreased with increasing axial length for measurements taken at or behind the equator. In a similar manner, scleral cross section area decreased marginally significantly with increasing axial lengths for the regions at or behind the equator (*P* = 0.002) (Fig. 3), at or behind the MPEPP (*P* = 0.001), and at or behind the optic nerve head border (*P* = 0.001). Scleral volume measurements were not significantly associated with axial length (Fig. 4).

The results of our study uniformly agree with previous investigations in that increasing axial length is associated with marked scleral thinning at and behind the equator [8]–[14]. The association between longer axial length and scleral thinning was the more pronounced the closer to the posterior pole. It suggested that the scleral changes associated with longer axial length take place predominantly in the posterior segment of the eye [8]–[11]. Associations between longer axial length and the cross-sectional area or the volume of the sclera had not systemically been addressed yet, so that the result of our study could not directly be compared with those of previous investigations.

Numerous previous studies on human globes as well as experimental studies on animals revealed that axial elongation is associated with a marked thinning of the sclera behind the equator of the globe, and these studies also addressed the role the sclera may play in the process of axial elongation, emmetropization and myopization [8]–[11], [14]–[37]. In a study by Avetisov and colleagues, scleral thickness changes from birth to adulthood were examined in emmetropic human eyes and in myopic human eyes [37]. Avetisov and coworkers found that the eye formation in ontogenesis was accompanied by a thickening of all scleral regions, in particular in the posterior region, by an accumulation of collagen and elastin in the posterior pole, by a reduced share of soluble collagen fractions, a lower content of glycosaminoglycanes in the equatorial region and an increase of tensile strength and elasticity module. The authors discussed that a group characterized by reduced content of collagen in the posterior scleral region, by a delayed decrease of the soluble collagen fractions with age in the posterior and equatorial regions and by a diminished tensile strength could be at risk for development and progress of myopia. Other experimental investigations showed that the myopia related increase in eye size resulted from an active scleral remodeling. This process included the production of a weakened scleral matrix [26]. McBrien and colleagues reported that within the first 24 hours of the development of myopia, the scleral elasticity was increased in animal eyes developing myopia [20,22.29]. The scleral creep rate (defined as tissue extension versus time) was also elevated in the sclera from eyes developing myopia, and reduced in eyes recovering from myopia. These myopic changes were thought to be due to changes in matrix constituents caused by scleral myofibroblasts. The latters are regulated by tissue stress and growth factors such as transforming growth factor-beta. Changes in these regulatory factors have been observed during myopia development, implicating cellular factors in the resultant weakened sclera. The findings of our study in association with the results of the previous investigation suggest that the whole process of active scleral remodeling as described in the experiments cited above does not lead to a major increase in scleral tissue volume.

The studies on human globes demonstrated that the scleral changes associated with long axial length were more marked the closer the measurement point was located to the posterior pole. It showed that the axial myopia associated thinning of the sclera was located predominantly posterior to the equator and increased in proximity to the posterior pole of the eye. Correspondingly, scleral thickness measurements at or posterior to the equator were not significantly correlated with corneal thickness measurements, fitting with clinical studies in which central corneal thickness was not related with axial length [38]. Since due to geometric reasons, the globe elongation is associated with an increase in scleral surface area, it was unclear whether despite the scleral thinning, the scleral cross sectional area and scleral volume also decreased or even increased. If the sclera is the primary site and tissue where the globe elongation is actively regulated, one may assume that the axial elongation due to an active process in the sclera is associated with an increase in scleral volume, masqueraded by a geometrically explained scleral thinning. Our study revealed however, that axial elongation was associated with a decrease in scleral cross sectional area in the region at or behind the equator, and that the scleral volume did not with longer axial length. It suggests that the active role of the sclera in the process of elongation of the eye does not include a marked increase in its tissue volume. These findings are supported by and confirm the results of a previous study by McBrien and colleagues who induced myopia in young tree shrews by monocular deprivation of pattern vision for short-term (12 days) or long-term (3–20 months) periods [22]. McBrien and associates observed a significant scleral thinning and scleral tissue loss, particularly at the posterior pole of the eye, in association with ocular enlargement and myopia development after both short- and long-term treatments. The collagen fibril diameter distribution was not significantly altered after short-term myopia treatment, whereas, from 3 months of monocular deprivation onward, significant reductions in the median collagen fibril diameter were noted, particularly at the posterior pole. The authors concluded that loss of scleral tissue and subsequent scleral thinning occurred rapidly during development of axial myopia, while an increased number of small diameter collagen fibrils in the sclera of highly myopic eyes was observed only in the longer term. These changes were considered to contribute to the weakened biomechanical properties of the sclera in highly myopic eyes. The decrease in scleral tissue as measured by weighing dried sclera in the study by McBrien may have an equivalent in the finding of our investigation in which the scleral volume measurements showed a slight tendency towards decreasing values with increasing axial length; these associations were however not statistically significant.

Potential limitations of our study should be mentioned. First, data on refractive error, corneal refractive power or corneal curvature radius were not available, so that the degree of hyperopia or myopia could not be determined. The study could therefore only refer to axial elongation as a surrogate for myopia or myopization. It may however be difficult for any histomorphometric study on human globes, in particular on a relatively large number of globes, to obtain reliable data on pre-enucleation refractive error. It may hold true in particular since most of the enucleated eyes had low vision or were blind. Second, due to postmortem swelling of the tissue after enucleation and due to the histological preparation of the slides, the measurements given in our study will not represent dimensions as determined *in vivo*. It was, however, not the purpose of the present investigation to evaluate the thickness of the sclera in real dimensions, but to assess associations between cross sectional area and volume of the sclera with axial length. Since the preparation induced changes in measurements affected the scleral measurements in a similar manner, the systemic error introduced by the histological preparation may not have affected the results of the statistical analysis. Third, our study included eyes with secondary angle-closure glaucoma or eyes enucleated due to a malignant melanoma. One may have to take into account that although myopic eyes and glaucomatous eyes have something in common in their scleral changes, they are not clearly interchangeable conditions. It has therefore remained unclear whether the results of our study can be generally transferred onto normal human eyes. Since congenital glaucoma was an exclusion criterion for our study, one may state that at least major scleral changes due to congenital glaucoma did not influence the results and conclusions of our study. One may also consider that it may be rather difficult or almost impossible to obtain normal human globes without post-mortem changes in sufficient number for a histomorphometric study. Fourth, serial sections of the globes were not available so that it was not possible to determine, whether the histological section was located in the very center of the optic disc or whether it ran slightly paracentrally. This limitation held true, however, for eyes with a normal axial length as well as for eyes with an abnormally long axial length. Fifth, the histological sections of the melanoma group were orientated according to the main location of the tumor, while the glaucomatous globes had been opened in a horizontal direction. Sixth, previous studies by McBrien and others have shown a loss of collagen fibril diameter gradients with scleral thinning. This observation was demonstrated in both human eyes and animal models of myopia development [22], [39], [40]. Since we did not apply electron microscopy in our study we could not address the question of an association in the scleral collagen fibril arrangement in association with axial elongation.

In conclusion, scleral cross-sectional area at or behind the equator decreased significantly with increasing axial length, and scleral volume was not statistically associated with axial length. It may suggest that, despite an associated increase in surface area, eyes with longer axial length did not show an increase in scleral volume. It points against a scleral volume enlargement in the role of the sclera in the process of axial elongation.
